# Relationship between adolescents’ family function with socio-demographic characteristics and behaviour risk factors in a primary care facility

**DOI:** 10.4102/phcfm.v2i1.177

**Published:** 2010-10-29

**Authors:** Abu S. Muyibi, Ike-Oluwapo O. Ajayi, Achiaka E. Irabor, Modupe M.A. Ladipo

**Affiliations:** 1 Family Medicine/General Outpatients Department, University College Hospital, Ibadan, Nigeria; 2 Department of Epidemiology, Medical Statistics and Environmental Health (EMSEH), University College Hospital, Ibadan, Nigeria

**Keywords:** Adolescents, behavioural risk factors, family function, relationships, primary care facility

## Abstract

**Background:**

The family as a unit of care has great effect in tackling adolescent problems and this could be influenced by family functioning.

**Objective:**

This study assesses the relationship between adolescents’ family functioning with socio-demographic characteristics and behavioural risk factors.

**Method:**

The research was a cross-sectional, hospital-based study carried out at the General Outpatients Department, University College Hospital (GOPD, UCH), Ibadan, over a period of three months. Four hundred subjects were recruited using a modified Guideline for Adolescent Preventive Services (GAPS) questionnaire, with an incorporated family APGAR (Adaptation, Partnership, Growth, Affection, Resolve) score table. The results were analysed using the Statistical Package for Social Sciences (SPSS), version 11 and the findings on the family assessment and behavioural risk factors were relayed to the respondents.

**Results:**

The ages of the adolescents ranged from 10 to 19 years. Of the subjects, 8% were sexually active. Mean age for first coitus among the respondents was 15 ± 2.4 years. The rate of ingestion of alcohol and cigarette smoking was very low. The family APGAR scores obtained revealed that 84.5% subjects were rated as having a functional family (7–10 points) and 15.5% of the subjects were rated as having a dysfunctional family (0–6 points). There was a significant association between perceived family function and subjects’ occupation (*p* = 0.01), parent social class (*p* = 0.00) and subjects’ sexual activities (*p* = 0.00).

**Conclusion:**

The majority of the adolescents were rated as having functional families. Dysfunctional families had significantly sexually active respondents.

## INTRODUCTION

The ‘age of adolescence’ has long been a fashionable phrase in the developed world, and has been recognised as a period of physical changes leading to ostensible physical maturity, poor judgement, risk-taking, strong peer influence and idealism.^1^

In Africa and Asia, adolescence is less spoken about, most likely as a result of economic and cultural factors.^2^ Adolescence, being a transition period from childhood to adulthood, is heralded by the onset of puberty.^1, 2, 3, 4, 5^ This stage in human development has been recognised as having a unique bio-psychosocial impact on the health of the individual. Adolescents constitute a group that is poorly identified in the health facilities of African countries and this has lead to their being grouped together with adults and thus, a denial of the particular, personalised care they deserve. To be able to address this, it is necessary to understand what adolescence is, and what age range it constitutes.^2, 6^

The definition of an adolescent varies from country to country. However, the World Health Organization (WHO) defines the adolescent as being a person between the age of 10 and 19 years, while youths are defined as persons between the age of 15 and 24 years.^2, 3, 4^ Nigeria's adolescent health policy has defined the adolescents age group as falling between the ages of 10 and 24 years.^2, 7^

The adolescent population is increasing worldwide and presently constitutes one-fifth (1.2 billion) of the world population.^2^ Four-fifths of adolescents live in developing countries, including Africa, where adolescents constitute about 30% of the total population.^1, 2^ In Nigeria, adolescents constitute about 30% of the total population, according to estimates made in 2006.^2, 3, 4^ With the increasing population of adolescents worldwide, more adolescents will be expected to present to the health care facilities with different illnesses. This is undoubtedly a large group that cannot be ignored or neglected in the health care scheme.

Health problems faced by an increasing number of adolescents from all sectors of the society include:

- psychosocial adjustment problems, and sexual and reproductive health problems such as sexually transmitted infections, including HIV and AIDS
- harmful traditional practices such as early marriage and female genital mutilation (FGM)
- alcohol and drug abuse
- accidental and unintentional injury
- endemic and infectious diseases
- nutritional problems
- mental health problems and eating disorders (depression, anorexia nervosa and bulimia); and
- dental health problems.^1, 2, 3, 5^

The presentation of these problems could be in the form of acute or chronic disease states,^6, 8^ and a fundamental emphasis and improvement on adolescent health care services are required, whereby a greater number of such services are directed at the primary and secondary prevention of these major health threats. The leading causes of morbidity and mortality in adolescents include trauma and drug abuse in developed countries,^1, 4, 6, 8^ while endemic diseases, unintended pregnancy and unsafe abortion are more common in developing countries.^2, 7, 9^ Adolescents can also be victims of sexual abuse or domestic violence. Pubertal growth problems and adolescent acne are unique disorders in adolescence, while dental caries is also a prominent disorder.^1, 2, 4, 6^ The various problems encountered in adolescents require visits to a health care facility.

Addictive behaviour is often referred to as ‘risk behaviour’, but it is a risk that adolescents are not proficient at assessing, since they do not understand the long-term consequences of adopting what they may regard as being only a temporary habit.^9, 10^ Alcohol consumption and drug use are considered risk behaviours because they reduce caution and impair judgement, thereby exposing the user to other risks and illegal habits. However, much of the adolescents’ behaviour is experimental and many adolescents pass through such periods unscathed.^9^

The WHO estimates that 500 million people who are alive today will eventually die of smoking-related diseases, including cancers, heart disease and respiratory diseases.^9^ Almost all regular smokers take up the habit by the age of 18. Cigarette smoking is one of the most common addictive behaviours amongst adolescents and this group is easy prey, since tobacco companies aim to recruit new smokers increasingly through catchy advertisements.^9^ As a result, worldwide mortality from tobacco smoking-related diseases is expected to rise to 10 million deaths a year by 2030, more than the total of deaths from malaria, maternal and major childhood conditions and tuberculosis combined.^9^ The most effective measures to prevent adolescents from taking up smoking in the first place include the placement of a ban on tobacco advertisements, increasing the price of tobacco products through taxation, and creating smoke-free areas in public places such as schools, colleges, health facilities and sporting venues.^9^

The major characteristics of growing up are exploratory and experimental behaviours that sometimes carry risks.^1, 2^ Some forms of sexual behaviour disorder, including sexual variation, sexual dysfunction, and sexual harassment or abuse, are all behavioural in origin.^2^ Premarital sex, early marriage and early childbearing are all interrelated.

In parts of Sub-Saharan Africa, almost half of the girls are pregnant by the age of 19, due to a legacy of unsafe or unprotected sex.^9^ Adolescent girls make up almost half of those having abortions, both outside or within marriage. Girls who become pregnant under the age of 18 are between two and five times more likely to die in childbirth than older women. Premarital sex and early marriage are often responsible for the unplanned or unwanted pregnancies and their consequences. Early marriage, resulting in sexual intercourse at a very young age, is sometimes defended on the grounds that it is a traditional cultural custom.^9^ While it is important for health services to be sensitive to cultural customs, this cannot take place at the expense of the health and well-being of vulnerable young people. The United Nation Convention on the Rights of the Child (UNCRC) is clear on this point. Article 24, which gives children and adolescents a right to health care, says in Clause 3: ‘States, parties shall take all effective and appropriate measures with a view to abolishing traditional practices prejudicial to the health of children’.^9^

Studies in several parts of Nigeria have shown that engaging in sex before marriage is relatively common, especially in urban areas.^2^ A study by Makinwa and Adebusoye in 1997 established that 8.4% of girls and 7.6% of boys had their first coitus between the ages of 12 and 14 years.^2, 11^ About a third of the girls and over half of the boys had had two to three partners. The majority did not use family planning methods regularly because of a lack of knowledge about contraception, and unsatisfactory service provision.^11^

In 1998, another study by Araoye and Fakeye on sexuality and contraception among Nigerian youths in Ilorin, Nigeria, found that 63% of the respondents under 20 years of age had had coitus, but only 11% and 22% of sexually active males and females, respectively, had ever used modern or traditional methods of family planning, despite the fact that they were relatively affordable.^12^ The same study also found that adolescents lacked knowledge of emergency contraception.^12^ The average number of sexual partners for both males and females was two, and the reasons for having sexual intercourse included, (1) the satisfaction of sexual desire, (2) the insistence of a boyfriend or girlfriend and (3) the satisfaction of financial needs. It was also noted that multiple sexual partners and unprotected coitus predisposed adolescents to sexually transmitted infections (STIs).^12^ Nigeria's overall STI rate is 16.5% and it is estimated that about 1 in 20 adolescents worldwide contract STIs annually.^13^

Adolescents may not present to their health care providers for routine health maintenance visits because they are generally healthy, and when sick, they fear stigmatisation and lack of confidentiality.^2^ In some cases, they do not have access to appropriate or affordable health care. This could be as a result of ignorance, the high cost of treatment, legal or cultural restrictions and the judgemental attitude of health workers. Health education is required to bridge this gap.^1, 2, 13^ When adolescents present for health care, it may be at the insistence of their parents or guardians, either voluntarily or by coercion.^4^ Other health care-seeking behavioural patterns of adolescents include being referred from school for medical certification of health, or they may be taken to health care facilities by law enforcement agents.^4, 6^ It has also been found that most of the morbidities experienced by adolescents are related to high-risk behaviours, which are preventable.^13^

The family as a unit of care has a great effect in tackling adolescent problems. Family ties are severely tried during the period when an adolescent is present. Families with adolescents can become closer, or conversely, more distant, when there are adolescent problems.^5^ The rapid changes in the family ties of kinship that bind individuals to their extended families have been further weakened in families with adolescents, possibly because of increasing urbanisation, which has adversely affected the adolescent in Nigeria and many other parts of the world.^5, 6^ Family systems theory defines the family as being an emotional unit.^14, 15^ When problems arise in the family, the ‘relationship systems’ carry more importance towards solutions than individual problem-handling measures, which supports the saying that ‘two heads are better than one’ in conflict resolution. Thus, families with adolescents, also known as crystallising family, according to Stevenson's family stage classification, need to be more flexible in order to accommodate the independence of the adolescents, and their need for autonomy.^16^ This may lead to conflicts if not properly managed.

The concept of family systems thinking and application was developed by Gabriel Smilkstein in 1968,^17^ and included physician attention to the systemic interactions of family members and the impact of conflicts, crisis, coping style and resources of family. He incorporated these components into the family APGAR tool (Adaptation, Partnership, Growth, Affection, Resolve), a simple instrument and mnemonic device for assessing the functioning of a family in health and illness (Appendix 1).

These APGAR statements focus on the emotional, communication, and social interactive relationships between the respondents and their families.^17, 18^

Family cohesion is conceptualised to include the degree of commitment, help and support that family members provide for one another. The levels of family cohesion have been implicated in both negative and positive health outcomes. High levels of family cohesion lead to bonding, and low levels of cohesion indicate poor family support, which could lead to a family dysfunction. Low family cohesion also results in poor individualisation and foreclosed adolescent psychosocial maturity, which are associated with poor disease control and a delay in accessing health care.^19, 20^

Family has been found to be a primary socialising agent and an expansive body of research has shown that adolescent risk behaviour is influenced by modifiable family influences, such as effective parenting (nurturing and supportive, with clear and consistent discipline). This prevents coercive family processes in early childhood, reinforces pro-social behaviour and facilitates child competencies that reduce the risk for problem behaviour in adolescence.^21^

Parental monitoring and supervision also prevents association with deviant peers, a primary pathway leading to onset and escalation of high-risk behaviour in adolescence. High levels of family conflict and poor family communication skills disrupt parenting and family relations, reduce children's emotional security and social-emotional competencies, and reinforce their use of aggression and interpersonal hostility. Family members also exert influence on adolescents through their own modelling of risk behaviours (deviance, substance use, aggression) and through shared core family processes.^21^

Dysfunction in a family occurs when there is a conflict, misbehaviour and even abuse on the part of individual family members continually, leading other members to accommodate such actions.^17, 18^ Common family dysfunction prototypes include family-head under-function, children being left alone to fend for themselves, and the inconsistency or violation of basic boundaries of appropriate behaviour. Dysfunctional family could stem from alcoholism or chronic health problems, the effect of which could pass down from generation to generation.^19, 20^

Family APGAR has been widely used to study the relationship of family and problems in family practice offices, but questions have arisen regarding the effectiveness of the family APGAR measure on family functioning. However in 1978, Smilkstein found that there were agreements between family APGAR scoring and clinician assessment.^20^ The family dynamics and level of family functioning have been found to influence adolescents’ risky behaviour, causation, progression and care of disease in the adolescents.^15^ This, therefore underscores the need for this study.

Family physicians (being frontline doctors), trained to appreciate the interaction of the family stage on the adolescent, and the effect of hormonal changes, mental changes and social influence, can easily manage common health situations occurring in adolescents. Research has shown that many adolescents prefer to present to the family physician for reproductive health problems among others,^5, 15^ which might be due to their identification of family physicians as being good communicators and strict observers of confidentiality.^15^

The findings of this study are also intended to inform family physicians about the need to assess family function upon first contact with adolescents.

### Objective

An assessment of the relationship between adolescents’ family functioning with socio-demographic characteristics and behavioural risk factors was carried out.

## ETHICAL CONSIDERATIONS

Ethical clearance was obtained from the University of Ibadan/University College Hospital (UCH) Joint Institutional Review Committee, and permission was also given by the head of the GOPD, UCH, Ibadan. Written, informed consent was obtained from each of the respondents or their guardian/parent. Assent was also obtained from the minors, in addition to consent from parents, before administration of the questionnaire used in the study and physical examination.

## METHODS

This study was conducted at the General Outpatients Department (GOPD) of the University College Hospital (UCH), Ibadan, south-western Nigeria, in the West African sub-region, from 1 February to 30 April 2007. The study population consisted mainly of Yorubas, who are the predominant ethnic group of this region.

The research was a cross-sectional, hospital-based study carried out at GOPD UCH in Ibadan. Sample size calculation was done using the sample size formula for descriptive studies:^22^ *n*=p(1-p)(Z/d)^2^, to arrive at minimum sample size of 400. Four hundred adolescents were systematically, randomly recruited, using a modified Guideline for Adolescent Preventive Services (GAPS) questionnaire, with an incorporated family APGAR score table.^6, 20^ The questionnaire was face-validated and pre-tested on 20 adolescents presenting at GOPD, UCH, Ibadan before the actual study was carried out, however the subjects involved in the pre-test were not included in the study itself. In addition to the recommended questions, information was also collected on adolescents’ social status, tobacco-smoking, alcohol consumption and ingestion of psychoactive stimulants, which may all contribute to family functionality.

The perceived family functioning was assessed with the family APGAR scale.^18^ This is a five-item validated scale of family functioning, developed to measure a family member's perception of the family function. The total score ranged from 0 to 10. The family APGAR score for each subject was calculated by summing the scores of the five items on the scale: the higher the score, the higher the level of perceived functionality of the family (Appendix 1). The 3-point scale was interpreted as, (1) ‘functional family’ (7–10 points), (2) ‘moderately dysfunctional family’ (4–6 points) and (3) ‘severely dysfunctional family’ (0–3 points). For the purpose of this study, the 3-point family APGAR scale was dichotomised into two categories, these being ‘functional family’ (7–10 points) and ‘dysfunctional family’ (0–6 points), when testing the association of subjects’ family function with socio-demographic characteristics.

The adolescents’ social status was determined by allocating them into their parents’ or guardians’ social classes, since they were still dependent. Parents’ social classification was done according to their occupation level at the time, based on the British Registrar-General classification.^23^ Though most of the respondents were students, the occupations of the few working adolescents were also noted. The adolescents’ parents’ social classification was as follows:

- Class I (professionals) was allocated to lawyers, doctors, accountants, and similar professionals.
- Class II (intermediate) was allocated to senior public servants, senior school teachers, nurse, and managers.
- Class III (skilled, non-manual) was allocated to junior school teachers, shop assistants, artisans and typists.
- Class IV (partly skilled, manual) was allocated to farm workers, drivers and bus conductors.
- Class V (unskilled, manual) was allocated to housewives, petty traders, cleaners, labourers and similar occupations.

The results were analysed using Statistical Package for Social Sciences (SPSS), version 11. The chi-square test was used to test for associations, and the level of statistical significance was set at *p* < 0.05.

## RESULTS

The ages of the adolescents ranged from 10 to 19 years, with a mean age of 13.5 ± 2.8 years for male (*n* = 206) and 14.4 ± 2.9 years for female subjects (*n* = 194). Of the respondents, 32 (8%) were sexually active, out of which 15 (3.8%) had had STIs. The mean age for first coitus was 15 ± 2.4 years. Seven of the subjects (6.2%) had been pregnant before, while four (3.5%) had had an abortion once in the past. Only three (1.6%) female subjects were married. The subjects’ mean age at first coitus among the 32 sexually active respondents in this study was 15.1 ± 2.4 years. Ten (31.3%) sexually active subjects were male and 22 (68.7%) subjects were female. The majority of the subjects (87.5%) had a single partner, while only a few (12.5%) respondents had two partners each. Only six (18.8%) of the 32 sexually active subjects had ever used barrier contraception (condoms). The prevalence of adolescent risky behaviour was low in this study. The ingestion of alcohol was noted as being 0.8% in the respondents, and the smoking of cigarettes was also noted as being 0.8%.

### Family APGAR rating

**FIGURE 1:**
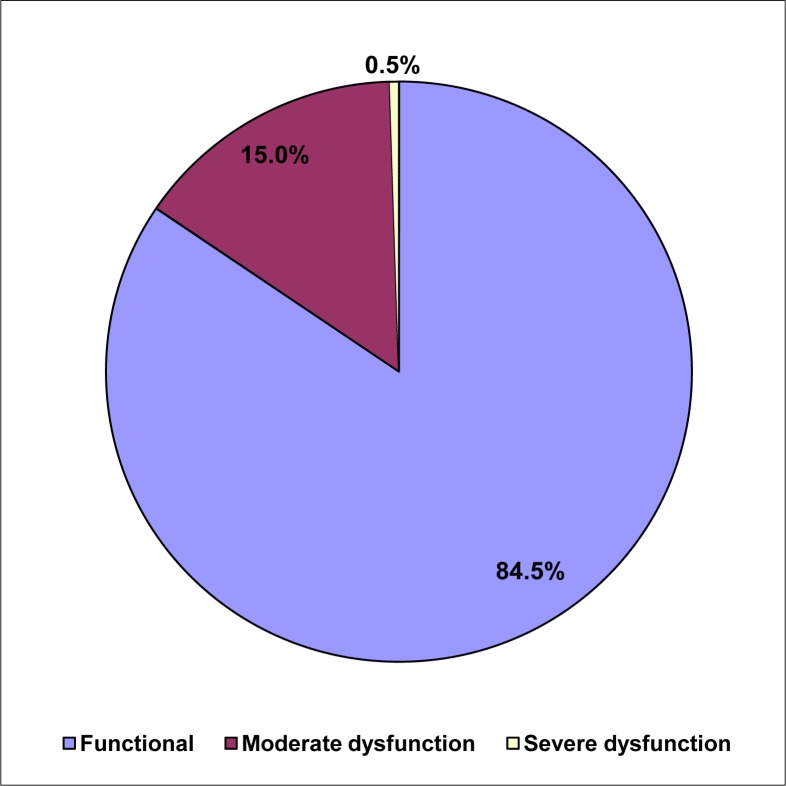
Adolescent family Adaptation, Partnership, Growth, Affection, Resolve rating

The range of family APGAR scores obtained was 3–10 points. The mean APGAR score was 7.94 ± 1.5 points. Of the adolescents in the study, 338 (84.5%) were part of a functional family (7–10 points). Sixty subjects (15%) belonged to moderately dysfunctional family (4–6 points), while two subjects (0.5%) were from severely dysfunctional family (0–3 points), as shown in Figure 1.

### Subjects’ family characteristics

**FIGURE 2:**
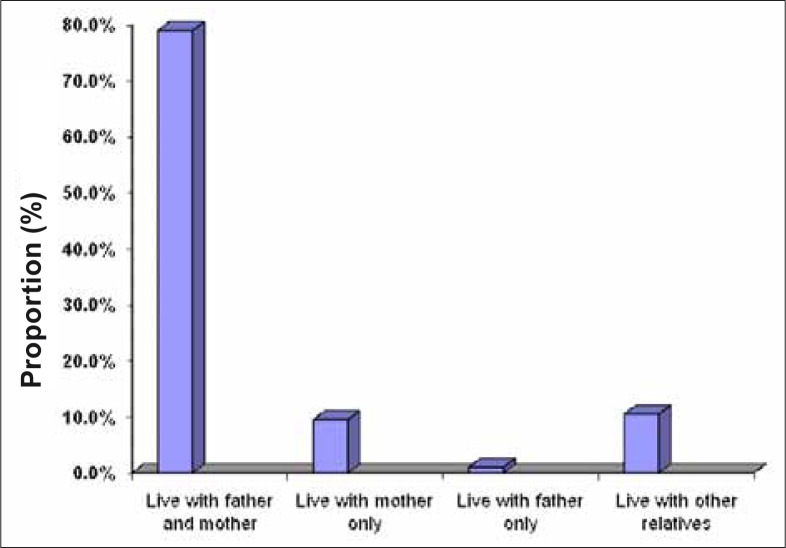
Adolescents’ living characteristics

An assessment of family characteristics showed that 79% of the adolescents lived with parents from whom they derived support (Figure 2).

A large percentage (82.5%) of the respondents’ parents were happily married (Table 1).

**TABLE 1:** Percentage distribution of subjects’ family characteristics (*n*) within the total population (*n* = 400)

Family characteristics	*n*	%
Respondent happy at home	371	92.8
Parents happily married	330	82.5
Parents divorced or separated	53	13.2
Parents/guardian meets financial needs	315	78.8
Respondent receive close supervision	301	75.2

### Association of subjects’ family function with socio-demographic characteristics

The social characteristics of the respondents studied showed that there was a significant association between perceived family function and subjects’ gender (*p* = 0.01), subjects’ occupation (*p* = 0.01), subjects’ parents’ social status (*p* = 0.00) and subjects’ sexual activities (*p* = 0.00); that is, the proportion of sexually active respondents was higher in dysfunctional families. For the purpose of testing the association of subjects’ family function with socio-demographic characteristics, the 3-point family APGAR scale was dichotomised into two categories: (1) functional family (7–10 points) and (2) dysfunctional family (0–6 points), as shown in Table 2.

**TABLE 2:** Association of subjects’ family function with socio demographic characteristics

Socio- demographic characteristics	Subject family function (APGAR score)					

	Dysfunctional (0–6 points)		Functional (7–10 points)		Total	
		
	*n*	%	*n*	%	*n*	%
**Gender†**						
Male	23	37.1	183	54.1	206	51.5
Female	39	62.9	155	45.9	194	48.5
**Total**	**62**	**100.0**	**338**	**100.0**	**400**	**100.0**
**Age group (years)‡**						
10–14	32	51.6	214	63.3	246	61.5
15–19	30	48.4	124	36.7	154	38.5
**Total**	**62**	**100.0**	**338**	**100.0**	**400**	**100.0**
**Occupation§**						
Schooling	56	90.3	331	97.9	387	96.7
Others	6	9.7	7	2.10	13	3.3
**Total**	**62**	**100.0**	**338**	**100.0**	**400**	**100.0**
**Parents social class¶**						
I	1	1.6	43	12.7	44	11.0
II	3	4.8	116	34.3	119	29.8
III	13	21.0	63	18.6	76	19.0
IV	40	64.5	111	32.8	151	37.7
V	5	8.1	5	1.6	10	2.5
**Total**	**62**	**100.0**	**338**	**100.0**	**400**	**100.0**
Subjects sexual activity**						
Sexually active	12	19.4	20	5.9	32	8.0
Sexually inactive	50	80.6	318	94.1	368	92.0
**Total**	**62**	**100.0**	**338**	**100.0**	**400**	**100.0**

* Significant at 5% level.

*n*, number of respondents affirmative; df, degree of freedom; χ^2^, chi square.

† χ^2^ = 6.08, df = 1, *p* = 0.01;

‡ χ^2^ = 3.02, df = 1, *p* = 0.08;

§ χ^2^ = 9.62, df = 1, *p* = 0.01;

¶ χ^2^ = 44.34, df = 4, *p* < 0.00, Fisher's exact test χ^2^ = 47.43, *p* = 0.00;

** χ^2^ = 12.82, df =1, *p* = 0.00

## DISCUSSION

The prevalence of adolescent risk-taking behaviour was low in this study. The consumption of alcohol was noted in 0.8% of the respondents and cigarette smoking was also noted in 0.8% of the adolescents. This is because most of the subjects involved in this facility-based study were school-going adolescents and mostly from functional families. This observation is supported by Aspy et al. in their 2006 study, which revealed a high quality of adolescent family relationship positively influencing risk behaviour characteristics.^24^

Of all the adolescents studied, 8% were sexually active, while 18.8% of the sexually active respondents used condoms.

The 2003 study of Overturf on adolescent risk behaviour in the USA found that between 30% and 40% of the respondents reported having tried smoking and drinking alcohol.^25^ Fewer, about 17%, had tried marijuana and only 5% report having used other illegal drugs. A quarter of all 14-year-olds to 17-year-olds reported having had sex, and about 75% of them said they or their partner had used a condom the last time they had had sex.^25^

A risk behaviour study at the central municipality of Belgrade by Tripovic in 2004, on adolescents, also shows that 46% of the subjects smoked cigarettes, 40% drank alcohol and 14% abused drugs.^26^ Boys aged 15–16 years made up 45% of the total population studied.^26^ This research showed that a significant percentage of addiction illness exists in Belgrade adolescents and adoption of the risky behaviour noted could endanger the adolescents later in life.^26^ In 2004, Omigbodun and Babalola in Ibadan, Nigeria, also found that psychoactive substance misuse among Nigerian adolescents had an effect on their mental health.^10^

Igwe et al., in their 2009 study on socio-demographic correlates of psychoactive substance abuse among secondary school students in Enugu, Nigeria, showed that 33.7% of the respondents were substance abusers.^27^ Alcohol was most commonly abused (31.6%) while cannabis was the least abused (4.1%). Males consumed most psychoactive substances more frequently than females, and 75% of the students were involved in multiple substance abuse. The older students were more involved in multiple abuses than the younger students.^27^

Since substance use among adolescents impacts on their health, and leads to risky sexual behaviour and other injurious activities, there is a need for parents, school authorities and government to pay serious attention to this problem. Regular counselling in schools has been advocated to sustain the awareness of the consequences of substance abuse among adolescents.^27^

An assessment of family characteristics in this study established that 79% of the adolescents lived with both parents from whom they derived support, and the majority of the respondents’ parents were happily married. Parental monitoring has been found to be an important correlate of adolescent risk behaviour, and the ability to monitor behaviour can be reduced if only one parental figure lives with the adolescent. Those who live with two parents (biological, step-parents, other, or any combination thereof) are significantly less likely to engage in risk behaviours such as smoking, property damage, illegal drug use, or running away from home, as found by Overturf in 2003.^25^ It is possible that adolescents with no chores have more freedom to do as they please with no parental control, while those with daily chores may be helping to run the household because their parent(s) are occupied with work or other demands. It appears, then, that teenagers with no chores or high levels of chores may have less parental monitoring, and are significantly more likely to report risky behaviours.^25^

Family cohesion is conceptualised to include the degree of commitment, help and support family members provide for one another. The levels of family cohesion have been implicated in both negative and positive health outcomes.^19, 20^ High levels of family cohesion lead to bonding (functional family), and low levels of cohesion indicate poor family support, which could lead to family dysfunction, poor disease control and a delay in accessing health care.^20^

An association of the subjects’ family functioning and socio-demographic characteristics showed that the majority (84.5%) of the subjects studied were from a functional family, while 15.5% of subjects were from a dysfunctional family. Consumption of alcohol and cigarette smoking were not significantly associated with family function because of the few adolescents involved.

Of the respondents from dysfunctional families, 51.6% were in the early adolescent age group studied. In the subjects from functional families, 97.9% were at school and the respondents who were home helpers (9.7%) were from dysfunctional families. This showed that functional families have more respondents at school, which could result in a more stable society.

An association between subjects’ parents’ social class and family function revealed that few subjects (6.4%) in social class I and II belonged to a dysfunctional family, while many subjects (85.5%), in social class III and IV were from a dysfunctional family. This implies that economic power contributes to the functionality of the family and ensures resources for coping with family crises.^5, 20^

The association between subjects’ sexuality and family functioning showed that a number of the sexually active subjects (19.4%) were from a dysfunctional family, while few sexually active subjects (5.9%) were from functional family. This finding was statistically significant and suggests that the issue of sexuality being a part of adolescent developmental attributes could be present in both functional and dysfunctional families.^17^ Maintenance of stable functional family would help to reduce the adolescents’ risk behaviours.

Social science research has demonstrated that parental involvement affects adolescent behaviour, primarily through monitoring on the part of parents.^25^ Parents who spend more time supervising their children have children who engage in fewer risky behaviours.^25^ Previous research also indicates that the quality of the mother-daughter relationship influences the age at which teenage girls first engage in sex.^28^

Premarital sex may be responsible for the 3.8% of STIs and the few (6.2%) teenage pregnancies noted in this study. Globally, STIs affect 1 in 20 young people annually. Though genital problems like STIs are mostly curable disorders, adolescents usually leave them untreated because they fear attending a clinic and would prefer to keep their ‘shameful’ risk behaviour secret.^11, 12^ Consequences of untreated genital problems among adolescents could result in pelvic inflammatory disease, ectopic pregnancy, infertility and depression later in life.

The Nigerian Demographic Health Survey (NDHS) in 2003 revealed that 24.8% of males and 29.8% of females aged 15–19 years have had unprotected sex and this has been responsible for the high rate of teenage pregnancy among the adolescents.^3^ Complications from teenage pregnancy and childbirth have been reported as the leading cause of death in young women aged 15–19 years in developing countries.^12, 13^ This is because the teenagers are not physically ready for parenthood. Sexuality and family life education have been found to be very useful in preparing young people to prevent teenage pregnancy. If pregnancy does occur, such prior knowledge could also help in coping with parenthood.^13^ This could be achieved by giving health education and reproductive health counselling at every encounter with adolescents.

A review by Fatusi, in 2005, of the status of adolescents’ reproductive health in Nigeria, indicated a high level of involvement of adolescents in unprotected sexual practice, resulting in teenage pregnancy and early childbearing, with complications and sexually transmitted infections.^13^ Araoye et al. in 1998 also associated premarital sex in adolescents with low contraceptive usage and unwanted pregnancies.^12^ Pregnancy is often terminated through illegal abortion and this is usually accompanied by fatal complications.^1, 6, 12, 13^

In this study, the majority of sexually active subjects (68.7%) were female and most had single partners. These findings were in agreement with previous studies, which found that female adolescents’ sexual maturity occurs earlier than that of males because of their hormonal differences.^1, 2, 8^ Thus, the females usually commence intimate relationship earlier than their male counterparts, thereby being more exposed to the risk of premarital sex and teenage pregnancy, as seen in this study, where 6.2% subjects have been pregnant in the past, while 3.5% of these subjects have had an abortion once in the past. This indicates very low contraceptive usage among the sexually active adolescents.

Overall, one-third of females from developing countries give birth before the age of 20.^2, 12^ This ranges from 8% in the South East Asia to 55% in the West African sub-region. Complications of pregnancy and childbirth are the leading causes of morbidity and deaths in young women of 15–19 years in developing countries.^9^ This could be prevented by promoting abstinence in sexually inactive respondents, while sexually active subjects should be encouraged to use contraceptives.

Social characteristics of the respondents studied revealed that the majority were students (96.7%), unemployed and thus economically dependent. The subjects’ general and financial supports were mainly from their parents and guardians whose social classes were primarily low-income groups (social class III and IV). This supports the observation that 21.2% of the subjects’ parents or guardians in this study could not meet their financial needs. This has arisen from the economic collapse of the 1980s that has led to the pauperisation of the middle class in Nigeria.^3^ By 1999, the proportion of Nigerians living below the poverty line rose from 28% to 66%, as documented by the Federal Office of Statistics.^2, 3^ To date, the proportion of Nigerians living below the poverty line is still rising and this could cause family dysfunction.^3, 17^ The adolescents in this study were at a disadvantage because of their total dependence on parents or guardians who struggle to make ends meet economically. The adolescents, even when employed, are poorly remunerated or not paid at all because of their lack of skills.

This study also revealed that the majority of the adolescents (99.2%) were single. Only 1.6% of the females were married, belonged to the late adolescents’ age group, and were not students. Early marriage is not usually practised among school-going adolescents in the study area (south-western Nigeria), as opposed to northern region of Nigeria. Although the prevalence of married adolescents (1.6%) among the females in this study is less than Nigeria's prevalence of 25.9% married girls between the age of 15 and 19 years, it is still of concern. However, the country's figure is for all females in a community-based study (Nigeria Demographic and Health Survey data), while this study is hospital-based.^3^ The early marriages noted among a few respondents in this study could have predisposed them to sexual activity and consequent teenage pregnancy.

### Limitations

The cultural inclination towards protection of family deficiency diminishes the objectivity of the Family APGAR scoring evaluation.

## CONCLUSION

In this study, the majority of the adolescents were rated as having functional families, which might have been due to strong family interactions and support noted in the African family structure.

Dysfunctional families were noted to have a significant proportion of sexually active respondents. Therefore, provision of routine family functioning assessment and regular family counselling for dysfunctional families could possibly stem the trend. This would strengthen the case for an adolescent-friendly health care service that is being encouraged by World Health Organisation.^9^

## Appendix

**APPENDIX 1:** Family Function (Family APGAR) scale

Component/closed-ended question.	Almost always	Sometimes	Hardly ever
	(2 points)	(1 point)	(0 points)
**1. A = Adaptation:**			
I am satisfied with the advice and support that I receive from my family when something is troubling me.			
**2. P = Partnership:**			
I am satisfied with the way my family discusses items of common interest and shares problem-solving with me.			
**3. G = Growth:**			
The relationship between me and my family is cordial/friendly.			
**4. A = Affection:**			
I am satisfied with the way my family expresses affection and responds to my feelings such as anger, sorrow and love.			
**5. R = Resolve:**			
I am satisfied with the way my family and I are able to resolve our differences in opinion and arrive at solutions.			

**Total points**			

*Source*: Smilkstein G. Family crisis and Family function APGAR test; In Family Medicine, principle and practice. Taylor RB, editor, New York, 1978;234–241.
